# Population-based volume kinetics of crystalloids and colloids in healthy volunteers

**DOI:** 10.1038/s41598-019-55171-1

**Published:** 2019-12-09

**Authors:** Jung-Min Yi, Ji-Yeon Bang, Bohyun Choi, Changhun Cho, Yong-Hun Lee, Eun-Kyung Lee, Byung-Moon Choi, Gyu-Jeong Noh

**Affiliations:** 1 Department of Anesthesiology and Pain Medicine, Catholic Kwandong University International St. Mary’s Hospital, Incheon, Korea; 2 Department of Anesthesiology and Pain Medicine, Asan Medical Center, University of Ulsan College of Medicine, Seoul, Korea; 3 Department of Nursing, Graduate School and College of Nursing Science, KyungHee University, Seoul, Korea; 4 Department of Anesthesiology and Pain Medicine, Asan Medical Center, University of Ulsan College of Medicine, Seoul, Korea; 5 Department of Anesthesiology and Pain Medicine, Asan Medical Center, University of Ulsan College of Medicine, Seoul, Korea; 6 Department of Statistics, Ewha Womans University, Seoul, Korea; 7 Department of Anesthesiology and Pain Medicine, Asan Medical Center, University of Ulsan College of Medicine, Seoul, Korea; 8 Department of Anesthesiology and Pain Medicine and Department of Clinical Pharmacology and Therapeutics, Asan Medical Center, University of Ulsan College of Medicine, Seoul, Korea

**Keywords:** Phase I trials, Computational models

## Abstract

We characterized the volume kinetics of crystalloid solutions (Ringer’s lactate solution and 5% dextrose water) and colloid solutions (6% tetrastarch and 10% pentastarch) by nonlinear mixed-effects modeling in healthy volunteers. We also assessed whether the bioelectrical impedance analysis parameters are significant covariates for volume kinetic parameters. Twelve male volunteers were randomly allocated to four groups, and each group received the four fluid solutions in specified sequences, separated by 1-week intervals to avoid any carryover effects. Volunteers received 40 ml/kg Ringer’s lactate solution, 20 ml/kg 5% dextrose water, 1000 ml 6% tetrastarch, and 1000 ml 10% pentastarch over 1 h. Arterial blood samples were collected to measure the hemoglobin concentration at different time points. Bioelectrical impedance spectroscopy (BIS, INBODY S10, InBody CO., LTD, Seoul, Korea) was also carried out at preset time points. In total, 671 hemoglobin-derived plasma dilution data points were used to determine the volume kinetic characteristics of each fluid. The changes in plasma dilution induced by administration of crystalloid and colloid solutions were well-described by the two-volume and one-volume models, respectively. Extracellular water was a significant covariate for the peripheral volume of distribution at baseline in the volume kinetic model of Ringer’s lactate solution. When the same amount was administered, the colloid solutions had ~4 times more plasma expansion effect than did the crystalloid solutions. Starches with larger molecular weights maintained the volume expansion effect longer than those with smaller molecular weights.

## Introduction

A variety of fluids are administered in clinical settings to maintain stable vital signs and intravascular volume status. Crystalloid solutions are used in most situations, but in cases which volume expansion is necessary, colloid solutions are used^1^. Plasma volume expansion and interstitial edema following administration of crystalloid or colloid solutions can be quantified using volume kinetics, a method used to analyze the disposition of infusion fluids^2–4^. Moreover, population analysis can be used to measure the interindividual variability of volume kinetic parameters^4,5^. Accurate quantification of the distribution and elimination of crystalloids or colloids used in clinical practice would contribute to the development of a more effective fluid therapy strategy. The fluid administration strategy in surgical patients has evolved over time. In the past, it was common to use a liberal fluid regimen in which fluids were administered to reflect the 3rd space loss and the deficit from withholding food and/or fluid; until a few years ago, restrictive fluid regimen was predominantly used to minimize fluid accumulation in the interstitium^6^. However, a recent large-scale study showed that the restrictive regimen increases the risk of acute kidney injury while providing minimal clinical benefit^7^. Thus, it is expected that a quantitative analysis of fluid distribution may contribute to the development of an effective strategy for fluid administration in surgical patients.

Previous studies reported that patients receiving Ringer’s lactate solution showed fluid accumulation in the interstitium caused by distribution through the extracellular fluid space^4,8^. Bioelectrical impedance analysis (BIA), a widely-used method for assessing body composition^9^, may help guide fluid management^10^. Thus, it would be meaningful to determine whether BIA parameters including extracellular water (ECW) may act as covariates for explaining the interindividual variability of the peripheral compartmental volume of distribution.

We thus aimed to characterize the volume kinetics of crystalloid solutions (Ringer’s lactate solution and 5% dextrose water) and colloid solutions (6% tetrastarch and 10% pentastarch) by nonlinear mixed-effects modeling in healthy volunteers. We also assessed whether BIA parameters are significant covariates for volume kinetic parameters.

## Materials and Methods

### Investigation fluid

Ringer’s lactate solution (JW Pharmaceutical Co., Ltd, Seoul, Korea), 5% dextrose water (JW Pharmaceutical Co., Ltd), 6% tetrastarch (hydroxyethyl starch, HES 130/0.4; VOLULYTE, Fresenius Kabi AG, Bad Homberg, Germany), and 10% pentastarch (HES 250/0.45; PENTASPAN, Jeil Pharmaceutical CO., LTD, Seoul, Korea) were used in this study.

### Study population

The protocols of this study were approved by the Institutional Review Board of Asan Medical Center (2014-0452) and was registered on an international clinical trials registry platform (http://cris.nih.go.kr; KCT0001791, Date of registration: February 2, 2016). All methods were performed as per the relevant guidelines and regulations of the institution. Written informed consent was obtained from all volunteers. Twelve male healthy volunteers aged 20–40 years with a body mass index (BMI) of 18.5–25 kg/m^2^ were enrolled. All volunteers abstained from smoking and alcohol consumption for at least 3 days before the study.

### Study design

We performed a randomized, open-label, four-period, crossover study. Volunteers were randomly allocated to one of the four groups, each of which received the four fluid solutions in specified sequences, separated by 1-week intervals to avoid carryover effects. The sequences were as follows: group 1: Ringer’s lactate solution → 6% tetrastarch → 5% dextrose water → 10% pentastarch; group 2: 6% tetrastarch → 10% pentastarch → Ringer’s lactate solution → 5% dextrose water; group 3: 10% pentastarch → 5% dextrose water → 6% tetrastarch → Ringer’s lactate solution; group 4: 5% dextrose water → Ringer’s lactate solution → 10% pentastarch → 6% tetrastarch. The volunteers received a maximum amount of fluid that can be given per day: 40 ml/kg for Ringer’s lactate solution, 20 ml/kg for 5% dextrose water, 1000 ml for 6% tetrastarch, and 1000 ml for 10% pentastarch^11–13^.

### Study procedures

All volunteers fasted from midnight on the day of infusion and arrived at the operating room without premedication. Volunteers were asked to empty their bladder and retain the rest. A 16 G angiocatheter was inserted into a cephalic vein for fluid infusion. A 20 G angiocatheter was placed in the radial artery of the ipsilateral arm to enable frequent blood sampling. Each fluid was infused in ten equally-divided doses administered over a 1-h period by manual infusion. Volunteers were maintained in a supine position throughout the study. Bioelectrical impedance spectroscopy (BIS; INBODY S10, InBody CO., LTD, Seoul, Korea) was measured before fluid infusion, after the end of each divided dose, and at 5, 10, 15, 20, 30, 40, 60, 90, 120, and 180 min after the end of fluid infusion. Body fluid composition parameters (extracellular water [ECW], intracellular water [ICW], total body water [TBW], extracellular water to total body water ratio [ECW/TBW]) were measured as well. The cumulative urine output was recorded at 3 hours after the end of fluid infusion.

### Measurement of hemoglobin

A total of 14 arterial blood samples (1 mL each) were collected to measure the hemoglobin concentration and hematocrit at preset intervals: before fluid administration, before the administration of the 2^nd^, 4^th^, 6^th^, 8^th^, and 10^th^ divided dose, and 5, 10, 15, 20, 30, 60, 120, and 180 min after the end of the fluid infusion. Blood tests were conducted at the clinical laboratories of Asan Medical Center, which is certified by the College of American Pathologists and Korean Society for Laboratory Medicine.

### Hemoglobin-derived plasma dilution

The equation for hemoglobin-derived plasma dilution can be expressed as follows^2^:

1 $$\frac{{v}_{c}(t)-{V}_{c}}{{V}_{c}}=\frac{\frac{Hb}{Hb(t)}-1}{1-Hct}$$

where *v*_*c*_ is the size of the expanded central body plasma fluid space, *V*_*c*_ is the baseline plasma fluid space, Hct is the hematocrit, and Hb is the hemoglobin concentration in the whole blood at baseline or time (*t*).

### Population analysis

Population-based volume kinetic analysis was performed with NONMEM VII level 4 (ICON Development Solutions, Ellicott City, MD, USA). Plasma dilutions were fitted to one- or two-volume models using the ADVAN 13 subroutines and first-order conditional estimation with interaction.

The differential equation of the one-volume model is as follows^4^:

2 $$\frac{dv(t)}{dt}={k}_{i}-{k}_{b}-{k}_{r}\times (\frac{v(t)-{V}_{0}}{{V}_{0}})$$

where *k*_*i*_ is the infusion rate of fluid, *k*_*b*_ is the baseline fluid loss, including insensible loss, *k*_*r*_ is the elimination clearance, *v* is the expanded body fluid space at the central compartment, and *V*_0_ is the baseline fluid space at the central compartment.

The differential equation of the two-volume model is as follows^4^:

3 $$\begin{array}{c}\frac{d{v}_{c}(t)}{dt}={k}_{i}-{k}_{b}-{k}_{r}\times (\frac{{v}_{c}(t)-{V}_{c0}}{{V}_{c0}})-{k}_{t}\times (\frac{{v}_{c}(t)-{V}_{c0}}{{V}_{c0}})+{k}_{t}\times (\frac{{v}_{t}(t)-{V}_{t0}}{{V}_{t0}})\\ \frac{d{v}_{t}(t)}{dt}={k}_{t}\times (\frac{{v}_{c}(t)-{V}_{c0}}{{V}_{c0}})-{k}_{t}\times (\frac{{v}_{t}(t)-{V}_{t0}}{{V}_{t0}})\end{array}$$

where *k*_*t*_ is the distributional clearance, *v*_*c*_ and *v*_*t*_ are expanded body fluid spaces at the central and peripheral compartments, respectively, and *V*_*c*0_ and *V*_*t*0_ are baseline fluid spaces at the central and peripheral compartments, respectively. *k*_*b*_ was set to 0.8 mL/min without estimating^14^.

The inter-individual random variabilities of the volume kinetic parameters were estimated assuming a log-normal distribution. Diagonal matrices were estimated for the various distributions of η, where η represents the inter-individual random variability with a mean of zero and variance of ω^2^. Residual random variability was modeled using an additive error model. NONMEM computed the minimum objective function value (OFV), a statistical equivalent to the −2 log-likelihood of the model. An α level of 0.05, which corresponds to a reduction in the OFV of 3.84 (chi-square distribution, degree of freedom = 1, *P* < 0.05), was used to distinguish among hierarchical models^15^. The covariates analyzed included age, body weight, height, body mass index, ECW, ICW, TBW, ECW/TBW, systolic arterial pressure, diastolic arterial pressure, and mean arterial pressure. A nonparametric bootstrap analysis was carried out to internally validate the models (fit4NM 3.3.3, Eun-Kyung Lee and Gyu-Jeong Noh; http://cran.r-project.org/web/packages/fit4NM/index.html; last accessed: March 16, 2011)^4^. For each fluid group, 1000 datasets were simulated from the respective final volume kinetic model, and the 90% prediction intervals were compared numerically and visually with actual plasma dilution^4,16^. Deterministic simulations were performed to compare the volume expansion effects of the four different fluids using the estimated volume kinetic parameters of each fluid model.

### Statistical analysis

Statistical analyses were conducted using R (version 3.5.1; R Foundation for Statistical Computing, Vienna, Austria) or SigmaStat version 3.5 for Windows (Systat Software, Inc., San Jose, CA, USA) as appropriate. Data are expressed as mean ± standard deviation for normally distributed continuous variables, median (25−75%) for nonnormally distributed continuous variables, and counts and percentages for categorical variables.

## Results

Twelve volunteers participated in all periods of the current study, all of which were included in the analysis. Physical characteristics of the volunteers and amounts of fluid administered are summarized in Table 1. In one volunteer, the plasma dilution value at 115 min after the administration of 1000 mL of 10% pentastarch was 0.12; the plasma dilution values measured before (75 min) and after (175 min) that time were 0.43 and 0.32, respectively. There were no events that would significantly affect the plasma dilution value at that time; therefore, the value of 0.12 measured at 115 min was considered a measurement error and was excluded from the population analysis. In total, 671 hemoglobin-derived plasma dilution data points from 12 volunteers (Ringer’s lactate solution: 168, 5% dextrose water: 168, 6% tetrastarch: 168, 10% pentastarch: 167) were used to determine the volume kinetic characteristics of each fluid.

**Table 1:** Volunteer characteristics and the amounts of fluid administered.

Age, yr	21.5 (21–35)
Body weight, kg	67.5 (5.1)
Height, cm	175.4 (4.8)
BMI, kg/m^2^	21.8 (1.8)
**Amounts of fluid administered, ml**	
Ringer’s lactate solution	2700 (213)
5% dextrose water	1358 (124)
6% tetrastarch	1000
10% pentastarch	1000

Data are presented as median (range) or mean (SD), as appropriate. BMI: body mass index.

**Figure 1:**
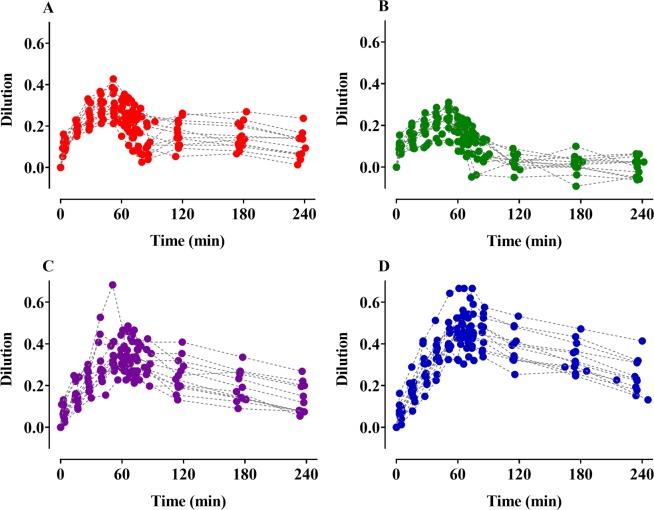
Time courses of plasma dilution during and after intravenous infusion over 1 h in 12 volunteers. (**A**–**D**) indicate Ringer’s lactate solution (40 mL/kg), 5% dextrose water (20 mL/kg), 6% tetrastarch (1000 mL), and 10% pentastarch (1000 mL), respectively.

**Figure 2:**
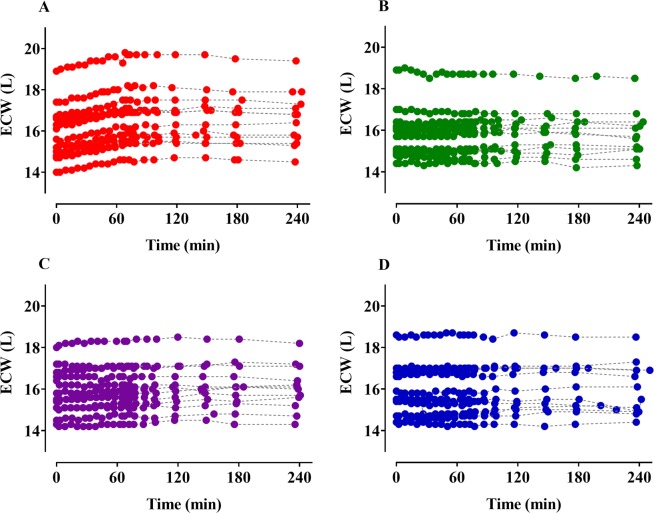
Changes of extracellular water (ECW) during and after intravenous infusion over 1 h in 12 volunteers. (**A**–**D**) indicate Ringer’s lactate solution (40 mL/kg), 5% dextrose water (20 mL/kg), 6% tetrastarch (1000 mL), and 10% pentastarch (1000 mL), respectively.

Time courses of plasma dilution (Fig. 1) and ECW (Fig. 2) during and after intravenous infusion of 4 different fluids over 1-h periods are shown. The dilutions increased to approximately 60 min after the start of administration of each fluid and then decreased. Although the colloid solutions were administered in lesser amounts than the crystalloid solutions, the degree of dilution was greater in the colloid solutions. Seven volunteers receiving 5% dextrose water showed negative plasma dilution values during the sampling period. The ECW gradually increased during the administration of Ringer’s lactate solution and remained almost unchanged in the remaining fluids. The two-volume model best described the volume kinetics of the crystalloid solutions, while the one-volume model well-depicted the time courses of plasma dilutions following colloid solution infusion. Table 2 shows the population volume kinetic parameter estimates and the results of non-parametric bootstrap replicates of the final models of the crystalloid and colloid solutions. ECW was a significant covariate for the peripheral volume of distribution at baseline (*Vt*_0_) (Eq. 4) in the volume kinetic model for Ringer’s lactate solution and resulted in improvement in the OFV (12.06, p < 0.05, degree of freedom (df) = 1) when compared with the basic model (number of model parameters = 8).

4 $$V{t}_{0}(L)=13.8+{16.8}^{(ECW/16)}$$

**Table 2:** Population-based volume kinetic parameter estimates, inter-individual variability, and median parameter values (2.5–97.5%) of the non-parametric bootstrap replicates of the final models for Ringer’s lactate solution, 5% dextrose water, 6% tetrastarch, and 10% pentastarch.

	Ringer’s lactate solution		5% dextrose water		6% tetrastarch		10% pentastarch	
	Estimates (RSE, %CV)	Median (2.5–97.5%)	Estimates (RSE, %CV)	Median (2.5–97.5)	Estimates (RSE, %CV)	Median (2.5–97.5%)	Estimates (RSE, %CV)	Median (2.5–97.5%)
*k*_*r*_ (L/min)	0.081 (22.5, 23.9)	0.078 (0.051–0.105)	0.425 (10.7, 32.7)	0.403 (0.268–0.511)	0.098 (13.1, 42.9)	0.097 (0.083–0.113)	0.038 (10.0, 30.5)	0.038 (0.033–0.043)
*Vc*_0_ (L)	4.9 (20.4, 22.6)	4.9 (2.9–6.1)	4.7 (15.4, 70.9)	3.7 (0.8–9.1)	16.3 (8.3, 29.3)	16.0 (14.3–17.7)	11.3 (6.8, 24.1)	11.2 (10.2–12.3)
*Vt*_0_ (L)	*θ*_1_ + *θ*_2_^(ECW/16)^ 13.8 + 16.8^(ECW/16)^ (*θ*_1:_ 41.4, 27.9) (*θ*_2:_ 29.3, 27.9)	*θ*_1:_ 13.5 (4.2–42.9) *θ*_2_: 17.2 (0.01–25.9)	5.4 (14.0, –)	5.6 (3.9–8.2)	—	—	—	—
*k*_*t*_ (L/min)	0.617 (3.9, –)	0.613 (0.554–0.653)	0.568 (31.3, –)	0.567 (0.161–0.960)	—	—	—	—
σ	0.0013	0.0013 (0.0011–0.0016)	0.0014	0.0014 (0.0012–0.0017)	0.0020	0.0021 (0.0017–0.0028)	0.0015	0.0015 (0.0012–0.0020)

A log-normal distribution of inter-individual random variability was assumed. Residual random variability was modeled using an additive error model. Non-parametric bootstrap analysis was repeated 2000 times. RSE, relative standard error = SE/estimate × 100 (%). ECW: extracellular water measured by bioimpedance spectroscopy, *k*_*r*_: elimination clearance, *Vc*_*0*_: central volume of distribution at baseline, *Vt*_*0*_: peripheral volume of distribution at baseline, *k*_*t*_: distributional clearance between central and peripheral compartments.

**Figure 3:**
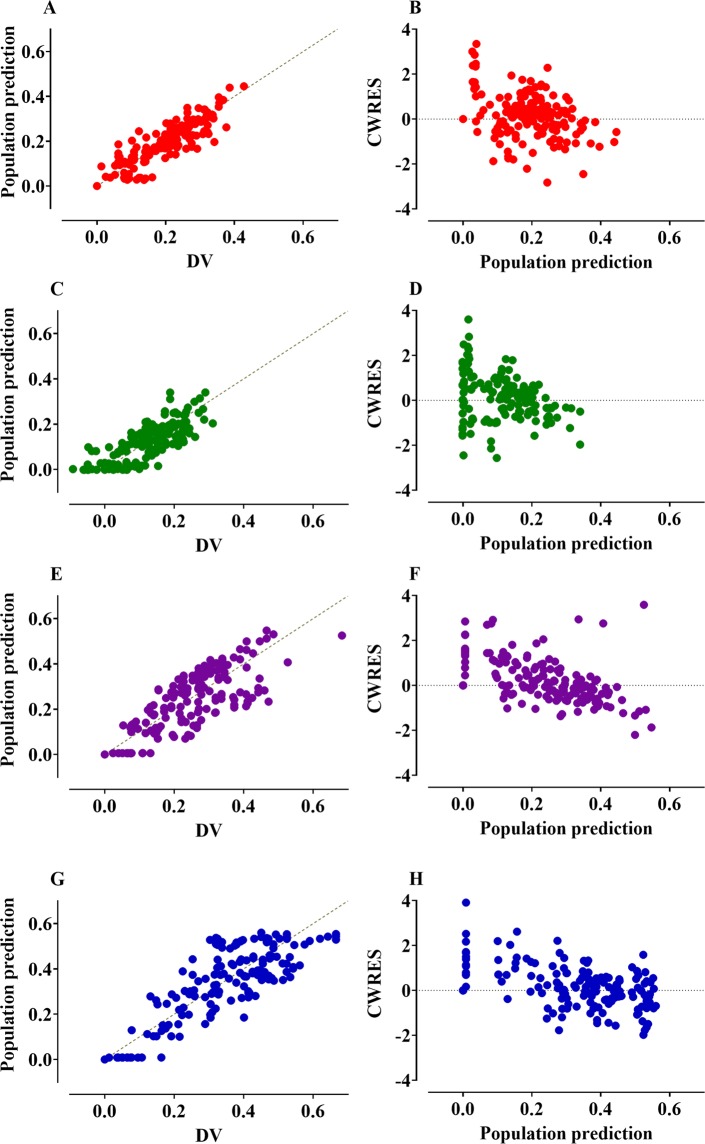
Goodness-of-fit plots of the final volume kinetic models. Ringer’s lactate solution (**A**,**B**), 5% dextrose water (**C**,**D**), 6% tetrastarch (**E**,**F**), and 10% pentastarch (**G**,**H**). (**A**,**C**,**E**,**G**) Population prediction *vs*. plasma dilution, (**B**,**D**,**F**,**H**) conditional weighted residuals (CWRES) *vs*. population prediction. The gold dashed lines indicate the lines of identity.

**Figure 4:**
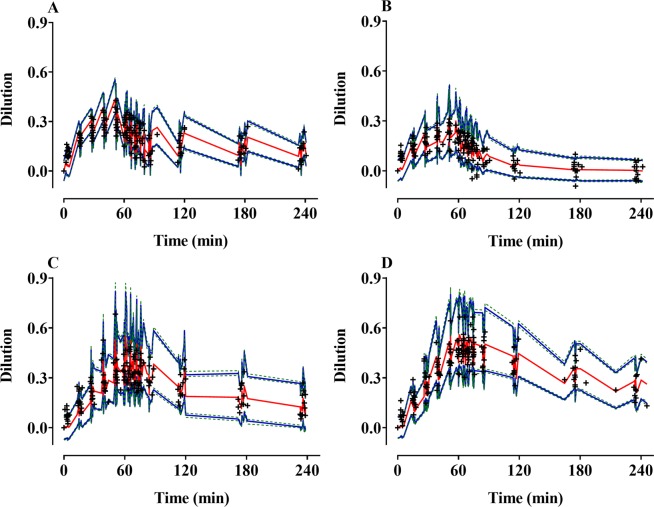
Predictive checks of the final volume kinetic models for Ringer’s lactate solution (**A**), 5% dextrose water (**B**), 6% tetrastarch (**C**), and 10% pentastarch (**D**). The red solid line indicates the 50% prediction line. The blue solid lines indicate the 5% and 95% prediction lines. The green dotted lines show 95% confidence intervals of the 5% or 95% prediction lines. + : observed dilution.

**Figure 5:**
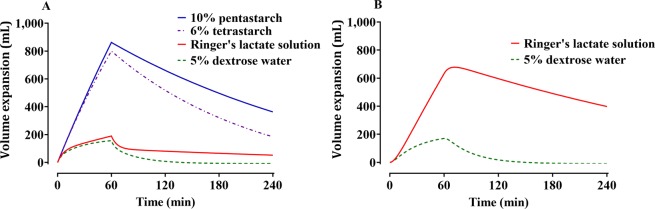
Simulated volume expansions of the central fluid space (**A**) and peripheral fluid space (**B**) in a hypothetical volunteer receiving 1000 mL of Ringer’s lactate solution, 5% dextrose water, 6% tetrastarch, or 10% pentastarch over 60 min. The extracellular water (ECW) of the hypothetical volunteer was set at 16 L.

The control streams of the final volume kinetic model that included ECW are presented in the Supplementary file. Goodness-of-fit plots of the final models are shown in Fig. 3. Predictive checks of the final models are shown in Fig. 4. The majority of the observed values were within the 90% prediction interval: 17.3%, 12.6%, 13.4%, and 13.6% of the data for Ringer’s lactate solution, 5% dextrose water, 6% tetrastarch, and 10% pentastarch, respectively, were distributed outside of the 90% prediction intervals of the predictive check. Figure 5 shows the simulated volume expansions of the central fluid space and peripheral fluid space in a hypothetical volunteer receiving 1000 mL of Ringer’s lactate solution, 5% dextrose water, 6% tetrastarch, or 10% pentastarch over 60 min. When the same amount was administered, the plasma volume expansion was more dominant in the colloid solutions than in the crystalloid solutions. Mean (SD) urine outputs during the entire sampling period were 1108 (305) mL, 1078 (390) mL, 636 (269) mL, and 580 (289) mL for Ringer’s lactate solution, 5% dextrose water, 6% tetrastarch, and 10% pentastarch, respectively. Urinary output was significantly higher in the crystalloid group than in the colloid group (one-way analysis of variance by Holm-Sidak post hoc test, Ringer’s lactate solution *vs* 10% pentastarch [P = 0.002], Ringer’s lactate solution *vs* 6% tetrastarch [P = 0.005], 5% dextrose water *vs* 10% pentastarch [P = 0.003], 5% dextrose water *vs* 6% tetrastarch [P = 0.007]).

## Discussion

Our results show that the changes in hemoglobin-derived plasma dilutions induced by administration of crystalloid and colloid solutions were well-described by two-volume and one-volume models, respectively. Extracellular water measured by bioelectrical impedance spectroscopy was a significant covariate for the peripheral volume of distribution at baseline in the volume kinetic model for Ringer’s lactate solution.

Generally, crystalloid solutions are distributed mainly in extracellular spaces rather than in blood vessels^17^. Thus, a large amount of crystalloid solution administered for intravenous fluid resuscitation can lead to accumulation in the interstitium and result in edema^17^, a phenomenon that was also observed in this study. At the end of the administration, only ~20% of the Ringer’s lactate solution remained in the blood vessels, and most of the solution was distributed in the extracellular space (Fig. 5). In the case of crystalloid solutions, the OFV of the two-volume model was lower than that of the one-volume model (OFVs of one-volume model and two-volume model: Ringer’s lactate solution: −700.89 and −900.45, respectively; 5% dextrose water: −871.93 and −881.47, respectively). In contrast, dextrose is rapidly taken up by cells and distributed in the intracellular space, with only a small amount remaining in the extracellular space^17^, which was also be observed in this study (Fig. 5). In terms of volume kinetic model parameters, the elimination clearance (*k*_*r*_) of 5% dextrose water was estimated to be much larger than those of other fluids (Table 2).

Colloid solutions, which contain both large organic macromolecules and electrolytes, are known to be effective volume expanders because they mainly stay in the blood vessels^17^. In fact, in the volume kinetic model for colloid solutions, OFV of the two-volume model was lower than that of the one-volume model (OFVs of one-volume model and two-volume model: 6% tetrastarch: −818.13 and −898.18, respectively; 10% pentastarch: −852.20 and −870.54, respectively). However, when the two-volume model was used, the central volume of distribution at baseline (*Vc*_0_) was estimated to be less than 1 L (6% tetrastarch: 0.97 L; 10% pentastarch: 0.33 L). *Vc*_0_ can be interpreted as anatomically comparable to the plasma volume prior to administration of each fluid. Plasma volumes in healthy male adults were reported to be approximately 47–52 mL/kg^18^, and because the estimated value of *Vc*_0_ should be at least 2 L, *Vc*_0_ was estimated as 2 L if the lower boundary of *Vc*_0_ was set to 2 L in the control stream. Therefore, we could not adopt the two-volume model to explain the plasma dilution induced by administration of colloid solutions. In previous studies, the volume kinetics of colloid solutions were also described through the one-volume model^2,19^.

In the volume kinetic model of Ringer’s lactate solution, it is physiologically logical that the peripheral volume of distribution at baseline (*Vt*_0_) increase as ECW increases. The increase in the ECW according to fluid administration can be explained by the increase in the expanded body fluid spaces at the peripheral compartments. The reason for the selective increase in the ECW in only in the Ringer’s lactate solution group may be that the actual amount of administered solution was considerably larger than other solutions (Fig. 2 and Table 1). In average adult males (weight: 65 kg; height: 175 cm), the predicted values for the 10^th^–90^th^ percentile of ECW was reported as 14.8–19.8 L^20^. Thus, 1 L of administered fluid corresponds to ~6% of ECW. In an animal study that aimed to develop an algorithm for estimating body composition, 20 ml of normal saline was administered to a 300 g rat to induce a change in ECW^21^, suggesting that it is difficult to measure the changes in ECW with BIA when the amount of fluid administered, is small. Accordingly, it seems that ECW was not a significant covariate for the volume of distribution in the remaining fluids. Likewise, the values of ICW and TBW were almost unchanged during and after intravenous infusion of 5% dextrose water (Supplementary Fig. S1).

The estimated elimination clearance (*k*_*r*_) in healthy volunteers was reported to be approximately 0.06–0.11 L/min^2,14^, which are in line with the *k*_*r*_ measured in this study. However, the *k*_*r*_ value in this study was lower than that estimated in a previous study conducted on patients undergoing open gastrectomy^4^. This difference may be explained in part by the following factors: anesthetic agents used for general anesthesia, types of surgery, patient’s physical characteristics, and volume status at the time of fluid administration. The estimates of central (*Vc*_0_) and peripheral (*Vt*_0_) volumes of distribution at baseline were also considered to be physiologically acceptable. Indeed, *Vt*_0_ was estimated to be somewhat larger than those in other studies^14,22^, which may be the main reason that distributional clearance between central and peripheral compartments (*k*_*t*_) was estimated to be high. the higher *k*_*t*_ values may be explained by the fact that volunteers were healthy young males and that their blood pressures were well-maintained during the study period. Considering the proportion occupied by the anatomical space outside the vessel^17^, the estimation of *Vt*_0_ at about 30 L is not likely to be a wrong estimate.

Theoretically, a crystalloid solution should have one-fourth the volume-expanding capacity as the same volume of colloid solution^23,24^. In one study, rapid infusion of the colloid solution was more effective than a crystalloid solution in increasing the blood volume in moderate hypovolemia^1^. Accordingly, our findings showed that at the end of the fluid administration, the colloid solutions showed about four-times higher efficacy in volume expansion in the central compartment compared with the crystalloid solutions (Fig. 5), even though there were no hypovolemic conditions such as bleeding. In our study, 10% pentastarch appeared to have a long-lasting volume expansion as compared to the 6% tetrastarch (Fig. 5); 10% pentastarch also had greater individual maximal dilution than did 6% tetrastarch (0.48 ± 0.09 *vs* 0.36 ± 0.08, paired t-test, P < 0.001). Besides, the elimination clearance (*k*_*r*_) of 10% pentastarch was smaller than that of 6% tetrastarch (Table 2). A previous study showed that 6% hydroxyethyl starch had larger *k*_*r*_ than did the 10% solution^25^. Although the blood collection required for dilution calculation did not last for a sufficiently long period until the volume expansion effect disappeared, 10% tetrastarch can be expected to have a longer volume expansion effect than 6% tetrastarch. In one study using the ^51^Cr radio-labelled red blood cell dilution technique, the effect of volume expansion was greater in the large molecular weight starch than in the smaller one until 12 hours after the end of fluid infusion^26^. To the best of our knowledge, only a few studies administered different crystalloid solutions and colloid solutions in volunteers with a cross-over design and quantified the distribution of these fluids by population analysis. It was also confirmed that in most patients, the ECW measured by BIA did not significantly change in response to administration of the usual amount of fluids. Our current results show that colloid solutions are more suitable than crystalloid solutions for increasing the plasma volume; crystalloid solutions required four-times the volume of colloid solutions for achieving the same amount of increase in plasma volume, and only about 20% of the amount administered remained in the blood space afterward.

## Conclusion

Our data show that the changes in hemoglobin-derived plasma dilutions in healthy male volunteers induced by administration of crystalloid and colloid solutions were well-described by the two-volume and one-volume models, respectively. A larger ECW as measured by BIA was associated with a greater peripheral volume of distribution at baseline. When the same amount was administered, the colloid solutions had about 4 times greater plasma expansion effect than did the crystalloid solutions. Starches with larger molecular weights maintained the volume expansion effects longer than those with smaller molecular weights.

## Supplementary information

Supplementary material

## Data Availability

The datasets generated during and/or analyzed during the current study are available from the corresponding author on reasonable request.
