# Supplementary material for: Population-based volume kinetics of crystalloids and colloids in healthy volunteers
Source: Sci Rep. 2019 Dec 9;9:18638. doi: 10.1038/s41598-019-55171-1 (PMC6901468; doi:10.1038/s41598-019-55171-1)
Supplement: Supplementary file 1 — Supplementary material [file 41598_2019_55171_MOESM1_ESM.pdf]

Population-based volume kinetics of crystalloids and colloids in healthy volunteers

Jung-Min Yi[1], Ji-Yeon Bang[2], Bohyun Choi[3], Changhun Cho[4], Yong-Hun Lee[5], Eun-Kyung Lee[6], Byung-Moon Choi[7], and Gyu-Jeong Noh[8]

1: Assistant professor, Department of Anesthesiology and Pain Medicine, Catholic Kwandong University International St. Mary's Hospital, Incheon, Korea (jminyi19@gmail.com)

2: Associate professor, Department of Anesthesiology and Pain Medicine, Asan Medical Center, University of Ulsan College of Medicine, Seoul, Korea (jyounbang@gmail.com)

3: PhD candidate, Department of Nursing, Graduate School and College of Nursing Science, KyungHee University, Seoul, Korea (equ194@naver.com)

4: Resident, Department of Anesthesiology and Pain Medicine, Asan Medical Center, University of Ulsan College of Medicine, Seoul, Korea (chochanghoon1@gmail.com)

5: Assistant professor, Department of Anesthesiology and Pain Medicine, Asan Medical Center, University of Ulsan College of Medicine, Seoul, Korea (yhlee0314@gmail.com)

6: Professor, Department of Statistics, Ewha Womans University, Seoul, Korea (lee.eunk@gmail.com)

7: Associate professor, Department of Anesthesiology and Pain Medicine, Asan Medical Center, University of Ulsan College of Medicine, Seoul, Korea (byungmoonchoi7@gmail.com)

8: Professor, Department of Anesthesiology and Pain Medicine and Department of Clinical Pharmacology and Therapeutics, Asan Medical Center, University of Ulsan College of Medicine, Seoul, Korea (nohgj@amc.seoul.kr)

Example of the control stream used in the volume kinetic model for Ringer's lactate solution.

```
$PROB RUN# 1112 (fluid kinetic model_HS_2COM)
$INPUT ID OID TIME AMT RATE DUR DV MDV SAMPLEID HB HCT PERIOD FLUID TYPE
AGE HT WT ICW ECW TBW ETRATIO HR SBP DBP MBP
$DATA 05_04_NONMEM_Bioimpedance_BP_HS.csv ACCEPT=(FLUID.EQ.1) IGNORE=#
$SUBROUTINE ADVAN13 TRANS=1 TOL=6
$MODEL COMP (VOLUME1) COMP (VOLUME2)
$PK
  TH1 = THETA(1)
  TH2 = THETA(2)
  TH3 = THETA(3)
  TH4 = THETA(4)
  TH5 = THETA(5)
  TH6 = THETA(6)

  KB = TH1 ; basal elimination rate (ml/min, 0.8 at Anesthesiology 1997;
87: 204-12)
  KR = TH2 ; renal clearance (ml/min)
  VC0 = TH3 ; baseline plasma volume (ml)
  VT0 = TH4 ; baseline interstitial volume (ml)
  KT = TH5 ; distributionl clerance  (ml/min)

  KB = TH1*EXP(ETA(1))
  KR = TH2*EXP(ETA(2))
  VC0 = TH3*EXP(ETA(3))
  VT0 = (TH4+TH6**(ECW/16))*EXP(ETA(4))
  KT = TH5*EXP(ETA(5))
```

```
$DES
  DADT(1) = RATE - KB - KR*(A(1)/VC0) - KT*(A(1)/VC0) + KT*(A(2)/VT0)
  DADT(2) = KT*(A(1)/VC0) - KT*(A(2)/VT0)

 ; A1: plasma volume expansion at central compartment (VC(t) - VC0); VC(t)
and VC0 mean plasma volume at any time and at baseline, respectively.
 ; A2: interstitial volume expansion at tissue compartment (VT(t) - VT0);
VT(t) and VT0 mean interstitial volume at any time and at baseline tissue
compartment, respectively.

$ERROR
  A1 = A(1)
  A2 = A(2)
  TA = A1/VC0
  TB = A2/VT0

  IPRED = TA
  W = 1
  IRES = DV - IPRED
  IWRES = IRES / W
  Y = IPRED + W * EPS(1)

  $THETA ; #5
  0.8 FIX ; KB
  (0, 20) ; KR
  (0, 2000) ; VC0
  (0, 6000) ; VT0
  (0, 100) ; KT
  (0, 10) ; VT0_ECW
 $OMEGA ; #3
```

```
  0 FIX ; IIV_KB
  0.3 ; IIV_KR
  0.3 ; IIV_VC0
  0.3 ; IIV_VT0
  0 FIX ; IIV_KT
 $SIGMA ; #1
   0.01

$ESTIMATION MAXEVAL=9999 SIGL=6 NSIG=2 PRINT=5 METHOD=1 INTER NOABORT
MSFO=1112.MSF
$COVARIANCE PRINT=E
```

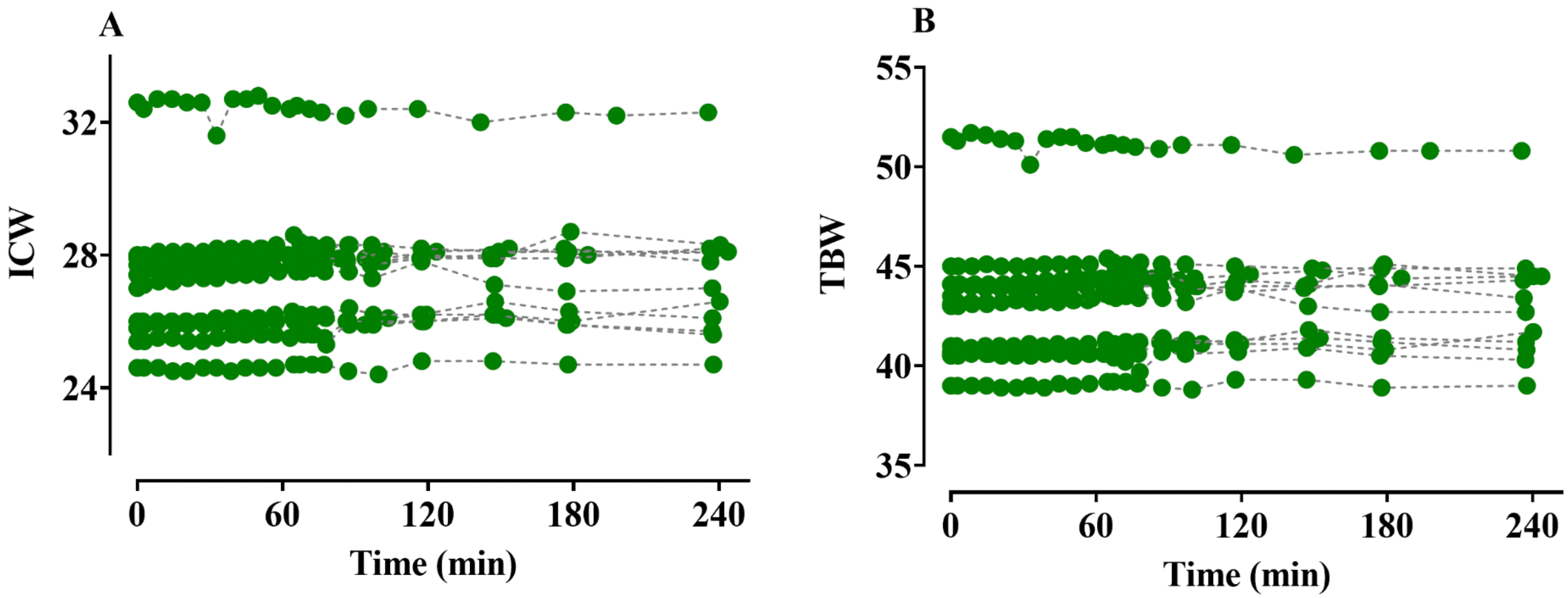

Supplementary figure. Changes of intracellular water (ICW; A) and total body water (TBW; B) during and after intravenous infusion of 5% dextrose water (20 mL/kg) over 1 h in 12 volunteers.
